# Supplementary material for: HIVprotI: an integrated web based platform for prediction and design of HIV proteins inhibitors
Source: J Cheminform. 2018 Mar 9;10:12. doi: 10.1186/s13321-018-0266-y (PMC5845081; doi:10.1186/s13321-018-0266-y)
Supplement: Supplementary file 2 — Additional file 2. Source code of HIVProtI web server. [file 13321_2018_266_MOESM2_ESM.zip › HIVprotI_Source-code/hivprotI/weka-3-6-6/doc/weka/filters/unsupervised/attribute/Standardize.html]

Standardize


---


|  |  |  |
| --- | --- | --- |
| **Overview** \| **Package** \| **Class** \| **Tree** \| **Deprecated** \| **Index** \| **Help** \| **Weka's home** | |  |
| **PREV CLASS**   **NEXT CLASS** | **FRAMES**    **NO FRAMES**     **All Classes** |  |
| SUMMARY: NESTED \| FIELD \| CONSTR \| METHOD | DETAIL: FIELD \| CONSTR \| METHOD |  |


---


## weka.filters.unsupervised.attribute Class Standardize

```
java.lang.Object
  weka.filters.Filter
      weka.filters.unsupervised.attribute.PotentialClassIgnorer
          weka.filters.unsupervised.attribute.Standardize
```

**All Implemented Interfaces:**: java.io.Serializable, CapabilitiesHandler, OptionHandler, RevisionHandler, Sourcable, UnsupervisedFilter

---

``` public class Standardize extends PotentialClassIgnorer implements UnsupervisedFilter, Sourcable ```

Standardizes all numeric attributes in the given dataset to have zero mean and unit variance (apart from the class attribute, if set).


Valid options are:

```
 -unset-class-temporarily
  Unsets the class index temporarily before the filter is
  applied to the data.
  (default: no)
```

**Version:**
:   $Revision: 5547 $

**Author:**
:   Eibe Frank (eibe@cs.waikato.ac.nz)

**See Also:**: Serialized Form

---

**Constructor Summary**

`Standardize()`


| **Method Summary** | |
| --- | --- |
| `boolean` | `batchFinished()`             Signify that this batch of input to the filter is finished. |
| `Capabilities` | `getCapabilities()`             Returns the Capabilities of this filter. |
| `java.lang.String` | `getRevision()`             Returns the revision string. |
| `java.lang.String` | `globalInfo()`             Returns a string describing this filter |
| `boolean` | `input(Instance instance)`             Input an instance for filtering. |
| `static void` | `main(java.lang.String[] argv)`             Main method for testing this class. |
| `boolean` | `setInputFormat(Instances instanceInfo)`             Sets the format of the input instances. |
| `java.lang.String` | `toSource(java.lang.String className, Instances data)`             Returns a string that describes the filter as source. |

**Methods inherited from class weka.filters.unsupervised.attribute.PotentialClassIgnorer**

`getIgnoreClass, getOptions, getOutputFormat, ignoreClassTipText, listOptions, setIgnoreClass, setOptions`

**Methods inherited from class weka.filters.Filter**

`batchFilterFile, filterFile, getCapabilities, isFirstBatchDone, isNewBatch, isOutputFormatDefined, makeCopies, makeCopy, numPendingOutput, output, outputPeek, toString, useFilter, wekaStaticWrapper`

**Methods inherited from class java.lang.Object**

`equals, getClass, hashCode, notify, notifyAll, wait, wait, wait`

**Constructor Detail**

### Standardize

```
public Standardize()
```


**Method Detail**

### globalInfo

```
public java.lang.String globalInfo()
```

:   Returns a string describing this filter

    :   **Returns:**: a description of the filter suitable for displaying in the explorer/experimenter gui

---


### getCapabilities

```
public Capabilities getCapabilities()
```

:   Returns the Capabilities of this filter.

    :   **Specified by:**: `getCapabilities` in interface `CapabilitiesHandler` **Overrides:**: `getCapabilities` in class `Filter`
    :   **Returns:**: the capabilities of this object **See Also:**: `Capabilities`

---


### setInputFormat

```
public boolean setInputFormat(Instances instanceInfo)
                       throws java.lang.Exception
```

:   Sets the format of the input instances.

    :   **Overrides:**: `setInputFormat` in class `PotentialClassIgnorer`
    :   **Parameters:**: `instanceInfo` - an Instances object containing the input instance structure (any instances contained in the object are ignored - only the structure is required). **Returns:**: true if the outputFormat may be collected immediately **Throws:**: `java.lang.Exception` - if the input format can't be set successfully

---


### input

```
public boolean input(Instance instance)
              throws java.lang.Exception
```

:   Input an instance for filtering. Filter requires all
    training instances be read before producing output.

    :   **Overrides:**: `input` in class `Filter`
    :   **Parameters:**: `instance` - the input instance **Returns:**: true if the filtered instance may now be collected with output(). **Throws:**: `java.lang.IllegalStateException` - if no input format has been set.: `java.lang.NullPointerException` - if the input format has not been defined.: `java.lang.Exception` - if the input instance was not of the correct format or if there was a problem with the filtering.

---


### batchFinished

```
public boolean batchFinished()
                      throws java.lang.Exception
```

:   Signify that this batch of input to the filter is finished.
    If the filter requires all instances prior to filtering,
    output() may now be called to retrieve the filtered instances.

    :   **Overrides:**: `batchFinished` in class `Filter`
    :   **Returns:**: true if there are instances pending output **Throws:**: `java.lang.Exception` - if an error occurs: `java.lang.IllegalStateException` - if no input structure has been defined

---


### toSource

```
public java.lang.String toSource(java.lang.String className,
                                 Instances data)
                          throws java.lang.Exception
```

:   Returns a string that describes the filter as source. The
    filter will be contained in a class with the given name (there may
    be auxiliary classes),
    and will contain two methods with these signatures:

    ```
     // converts one row
     public static Object[] filter(Object[] i);
     // converts a full dataset (first dimension is row index)
     public static Object[][] filter(Object[][] i);
    ```

    where the array `i` contains elements that are either
    Double, String, with missing values represented as null. The generated
    code is public domain and comes with no warranty.

    :   **Specified by:**: `toSource` in interface `Sourcable`
    :   **Parameters:**: `className` - the name that should be given to the source class.: `data` - the dataset used for initializing the filter **Returns:**: the object source described by a string **Throws:**: `java.lang.Exception` - if the source can't be computed

---


### getRevision

```
public java.lang.String getRevision()
```

:   Returns the revision string.

    :   **Specified by:**: `getRevision` in interface `RevisionHandler` **Overrides:**: `getRevision` in class `Filter`
    :   **Returns:**: the revision

---


### main

```
public static void main(java.lang.String[] argv)
```

:   Main method for testing this class.

    :   **Parameters:**: `argv` - should contain arguments to the filter: use -h for help


---


|  |  |  |
| --- | --- | --- |
| **Overview** \| **Package** \| **Class** \| **Tree** \| **Deprecated** \| **Index** \| **Help** \| **Weka's home** | |  |
| **PREV CLASS**   **NEXT CLASS** | **FRAMES**    **NO FRAMES**     **All Classes** |  |
| SUMMARY: NESTED \| FIELD \| CONSTR \| METHOD | DETAIL: FIELD \| CONSTR \| METHOD |  |


---
